# The Molecular Detection of Germline Mutations in the *BRCA1* and *BRCA2* Genes Associated with Breast and Ovarian Cancer in a Romanian Cohort of 616 Patients

**DOI:** 10.3390/cimb46050281

**Published:** 2024-05-12

**Authors:** Liliana-Georgiana Grigore, Viorica-Elena Radoi, Alexandra Serban, Adina Daniela Mihai, Ileana Stoica

**Affiliations:** 1 Doctoral School of Biology, Faculty of Biology, University of Bucharest, 030018 Bucharest, Romania; 2 Personal Genetics, 010987 Bucharest, Romania; 3 Department of Medical Genetics, “Carol Davila” University of Medicine and Pharmacy, 020021 Bucharest, Romania; 4 “Alessandrescu-Rusescu” National Institute for Maternal and Child Health, 20382 Bucharest, Romania; 5 Department of Genetics, Faculty of Biology, University of Bucharest, 030018 Bucharest, Romania

**Keywords:** breast cancer, ovarian cancer, *BRCA1* gene mutation, *BRCA2* gene mutation

## Abstract

The objective of this study was to identify and classify the spectrum of mutations found in the *BRCA1* and *BRCA2* genes associated with breast and ovarian cancer in female patients in Romania. Germline BRCA1 and BRCA2 mutations were investigated in a cohort of 616 female patients using NGS and/or MLPA methods followed by software-based data analysis and classification according to international guidelines. Out of the 616 female patients included in this study, we found that 482 patients (78.2%) did not have any mutation present in the two genes investigated; 69 patients (11.2%) had a *BRCA1* mutation, 34 (5.5%) had a *BRCA2* mutation, and 31 (5%) presented different type of mutations with uncertain clinical significance, moderate risk or a large mutation in the *BRCA1* gene. Our investigation indicates the most common mutations in the *BRCA1* and *BRCA2* genes, associated with breast and ovarian cancer in the Romanian population. Our results also bring more data in support of the frequency of the c.5266 mutation in the BRCA1 gene, acknowledged in the literature as a founder mutation in Eastern Europe. We consider that the results of our study will provide necessary data regarding *BRCA1* and *BRCA2* mutations that would help to create a genetic database for the Romanian population.

## 1. Introduction

The autosomal dominant inherited cancer predisposition characterized by an increased risk of breast and ovarian cancers is called hereditary breast and ovarian cancer syndrome (HBOC). This type of familial/hereditary cancer represents approximately 10–15% of all breast cancer and 25% of ovarian cancer cases [1]. Breast cancer has become the most commonly diagnosed cancer worldwide, overtaking lung cancer, with over 2.200.000 new cases in 2020 in both sexes. In Romania, female breast cancer had 12.085 cases, with a cumulative risk (0–74) of 10.3, and the number of prevalent cases (5 years) was 45,263 in 2020 [2]. Hereditary (familial) breast cancer represents only 10% of all breast cancer cases. Almost half of these are associated with mutations in *BRCA1* (BReast CAncer gene 1) and *BRCA2* (BReast CAncer gene 2) genes, while the other half are associated with mutations in different genes, such as *PALB2* (partner and localizer of *BRCA2*), *CHEK2* (checkpoint kinase 2), and *PTEN* (phosphatase and tensin homolog). Germline mutations in the *BRCA1* and *BRCA2* genes are reported in about 15–20% of all triple-negative breast cancers [3,4]. Ovarian cancer, despite being only eighth place in the number of new cases in 2020 with 313.959, is a silent public health concern due to its high mortality rate. In Romania, ovarian cancer had 1.909 cases with a cumulative risk (0–74) of 1.7, and the number of prevalent cases (5 years) was 5.302 in 2020 [2]. The most common subtype of epithelial ovarian cancer is the high-grade serous ovarian cancer (HGSOC) subtype, which is mostly associated with *BRCA1*/2 mutations [5,6,7] and, in some cases, with mutations in the *RAD51C* (*RAD51* Paralog C), *RAD51D* (*RAD51* Paralog D), and *BRIP1* (*BRCA1* Interacting Helicase 1) genes [8]. Patients with this subtype of ovarian cancer who are negative for somatic mutations of the *BRCA1* and *BRCA2* genes will be subsequently tested for HRD (Homologous Recombination Deficiency) score evaluation. If the HRD score is positive, these patients could benefit from targeted therapies, such as PARP (poly (ADP-ribose) polymerases) inhibitors (PARPi) [9,10]. Some of the major risk factors for developing breast or ovarian cancer consist of a family history of different types of cancer, the use of estrogen and progesterone replacement therapies, advanced age at first birth, exposure to radiation or other toxic environmental factors, and an unbalanced diet and lack of physical activity [11,12,13,14].

Carriers of *BRCA1* mutations have an estimated lifetime risk of developing breast cancer of 55–72%, a 39–44% risk of developing ovarian cancer, a 21–29% risk for males of developing prostate cancer, and a 1–2% risk for males of developing breast cancer, as well as a 1–3% chance of developing pancreatic cancer. These estimated lifetime risks may differ according to the location or type of mutation [15]. Carriers of *BRCA2* mutations have a 45–69% risk of developing breast cancer, an 11–17% risk of developing ovarian cancer, and a 27–60% risk for males of developing prostate cancer, as well as a higher *BRCA1* risk of 6–8% for males of developing breast cancer and a 3–5% risk of developing pancreatic cancer [10,16,17].

These two types of cancers (female breast and ovarian cancer) are mostly curable when they are discovered in the early stages of development and when medical treatment is available and affordable. Even though these types of cancer are treatable, chemoresistance and recurrence are very frequent and significantly worsen the prognosis of the disease. Genetic testing of the *BRCA1* and *BRCA2* gene mutations is particularly recommended for individuals with familial susceptibility to cancer or who have a personal history of cancer. In Romania, many patients are diagnosed only in the late stages of breast and/or ovarian cancer development due to a low level of population screening and prophylactic programs (a lack of funding and/or poor advertisement) to inform people about cancer risk [16,18,19,20,21,22].

Hopefully, to possibly add another tool in helping to reduce the diagnostic time for patients with breast and/or ovarian cancer, our study brings new information about the most frequent mutations of *BRCA1* and *BRCA2* genes associated with breast or ovarian cancer in the Romania population. Our data, along with those reported by other groups from different regions of Romania [23,24,25,26], could represent the start of the development of a national cancer-related database that would help improve the management of the patients.

## 2. Materials and Methods

Biological samples consisted of peripheral blood collected from 616 female patients (spanning from January 2019 to March 2022) diagnosed with breast and/or ovarian cancer who were referred to Personal Genetics, Bucharest, Romania for BRCA1/2 germline mutation status. The study was conducted in accordance with the Declaration of Helsinki and was approved by the Institutional Review Board (or Ethics Committee) of Personal Genetics (107/16 March 2022).The analyses were performed at NeoScreen Genetics Center, Greece, and all patients completed and signed a written informed consent/GDPR agreement.

Patient selection. For ovarian cancer, we included in our study 432 patients diagnosed with non-mucinous ovarian cancer, which represents one of the most common subtypes of ovarian cancer associated with BRCA 1/2 mutations [13,27]. Of the 432 patients, 390 had the high-grade serous ovarian cancer subtype and the remaining 42 had no defined subtype. For breast cancer, we included 184 patients who have been diagnosed with triple-negative breast cancer, a subtype of breast cancer that is characterized by having no expression of an estrogen receptor (ER), a progesterone receptor (PR), or human epidermal growth factor receptor 2 (HER2). This subtype is highly aggressive, has a shorter survival rate and a higher likelihood of recurrence, and was considered due to a higher association with BRCA1 gene mutations [20,28,29].

Total genomic DNA isolation from blood samples was performed using the “NucleoSpin Tissue kit” (Product Code 11982262; Macherey-Nagel, Thermo Fisher Scientific, Waltham, MA, USA), according to the manufacturer’s protocol.

The DNA sample was subjected to point mutation detection analysis (base substitutions and short deletions/duplications of a few base pairs), which was conducted through massively parallel sequencing of the coding regions of the *BRCA1* (NM_007294.4) and *BRCA2* genes (NM_000059.3), including exon–intron junction regions. Sequencing was performed using the “Accel Amplicon™ *BRCA1*, *BRCA2* panel” kit (Product code: AL-52048; Swift Biosciences, Ann Arbor, MI, USA) on a “Next Generation Sequencing” platform MiSeq (Illumina, San Diego, CA, USA). The detection of large genomic rearrangements (deletions/duplications of 1 or more exons) in the *BRCA1* and *BRCA2* genes was conducted by multiplex ligation-dependent probe amplification (MLPA) using the products P002-D1 and P045-D1 (MRC-Holland, Amsterdam, The Netherlands). Capillary electrophoresis was performed using the ABI3130xl genetic analyzer. All the SNP (single nucleotide polymorfism) mutations identified were confirmed ulteriorly through Sanger sequencing.

The analysis of the FASTQ files obtained from the massively parallel sequencing process on the MiSeq sequencing platform was performed with the following steps:

Quality assessments of the reads before alignment, which involve the exclusion of nucleotides/reads with low quality;

Alignment process performed using the BWM-MEM algorithm, thus generating the specific BAM files;

Quality control post alignment process and coverage analysis;

Detection of mutations (substitutions, small deletions, small insertions/duplication) based on the GATK (Genome Analysis Toolkit) algorithm and the characterization of the detected sequence variants using the following databases: ClinVar, OMIM (Online Mendelian Inheritance in Man), 1000GENOMES, GnomeAD (Genome Aggregation Database), Varsome, LOVD (Global Variome shared Leiden Open-source Variation Database), BIC, and INVITAE. If a missense mutation is detected, in silico analysis is performed using the dbNSFP program. The large deletions and/or large duplications were detected via bioinformatics analysis of raw data obtained by MLPA (Multiplex Ligation-dependent Probe Amplification) technology. MLPA data analysis was performed using Coffalyser Net software v.140721.1958 (MRC Holland, Amsterdam, The Netherlands).

## 3. Results

Our results show that out of the 390 patients diagnosed with non-mucinous ovarian cancer, the high-grade serous ovarian cancer subtype, 300 had a negative result, 48 had a pathogenic *BRCA1* mutation, 24 had a pathogenic *BRCA2* mutation, two had a mutation in the *BRCA1* gene with uncertain significance, six had a mutation in the *BRCA2* gene with an uncertain significance VUS (variant with uncertain significance), five had the moderate risk variant c.9976A>T (p.Lys3326Ter) in the *BRCA2* gene, and five had a large deletion in the *BRCA1* gene (Table 1).

Of the forty-two patients diagnosed with non-mucinous ovarian cancer with no defined subtype, thirty were negative, three had a pathogenic *BRCA1* mutation, six had a pathogenic *BRCA2* mutation, one had a VUS mutation in the *BRCA2* gene, and two had the moderate risk variant c.9976A>T (p.Lys3326Ter) in the *BRCA2* gene (Table 1).

From the 184 patients diagnosed with triple-negative breast cancer, 152 had a negative result, 18 had a pathogenic *BRCA1* mutation, four had a pathogenic *BRCA2* mutation, two had a VUS mutation in the *BRCA1* gene, two had a VUS mutation in the *BRCA2* gene, four had the moderate risk variant c.9976A>T (p.Lys3326Ter) in the *BRCA2* gene, and two had a large deletion in the *BRCA1* gene (Table 1).

### 3.1. Variants in the BRCA1 Gene

Regarding their clinical significance, in the *BRCA1* gene, the mutations were classified as pathogenic mutations (base substitutions and short deletions/duplications of a few base pairs—nineteen variants) (Table 2), VUS mutations (four variants) (Table 3), and large deletions of different regions with pathogenic significance (three variants). The three variants that affect large regions of the *BRCA1* gene were detected by MLPA, and they are heterozygous deletions of the upper region of exons 1 and 2, heterozygous deletion of exon 8, and heterozygous deletions of exons 21 and 22.

**Figure 1:**
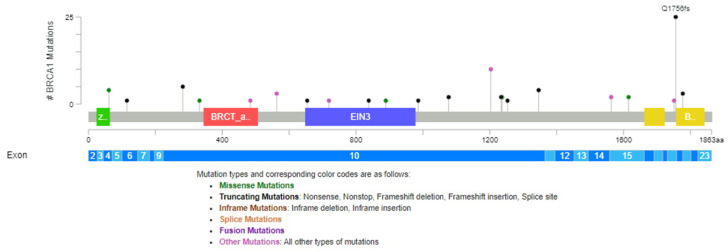
Distribution of *BRCA1* mutations across protein domains (green box—Zinc finger, C3HC4 type (RING finger) domain, red box—Serine-rich domain associated with BRCT, blue box—Ethylene insensitive 3, yellow—BRCA1 C Terminus (BRCT) domain).

The pathogenic and VUS mutations in the *BRCA1* gene found in our group study are represented across protein domains in Figure 1 using the cBio Cancer Genomics Portal [30,31,32].

### 3.2. Variants in the BRCA2 Gene

Regarding their clinical significance, in the *BRCA2* gene, the mutations were classified as pathogenic mutations (base substitutions and short deletions/duplications of a few base pairs—twenty-three variants) (Table 4), VUS mutations (eight variants) (Table 5), and one moderate risk variant c.9976A>T (p.Lys3326Ter), which is assigned as benign in the ClinVar database. Despite not being a pathogenic mutation, it has been previously shown in the literature that patients carrying this variant may be good candidates for PARP inhibitor therapy due to *BRCA2* protein dysfunction induced by the modification of this variant over the C-terminal region of the protein [33].

**Figure 2:**
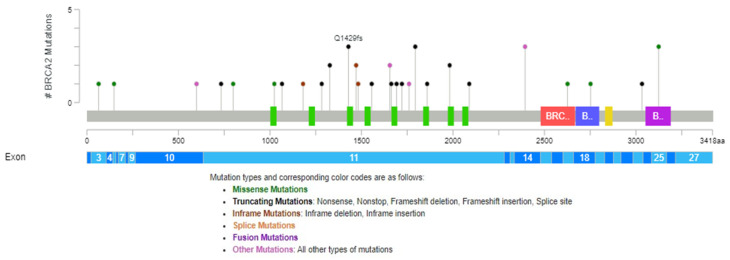
Distribution of *BRCA2* mutations across protein domains (green boxex—BRCA2 repeat, red box—BRCA2_helical, blue box—BRCA2, oligonucleotide/oligosaccharide-binding, domain 1, yellow box—Tower, purple box—BRCA2, oligonucleotide/oligosaccharide-binding, domain 3).

The pathogenic and VUS mutations in the *BRCA2* gene found in our group study are represented across protein domains in Figure 2 using the cBio Cancer Genomics Portal [30,31,32].

## 4. Discussion

Our study results identified three main pathogenic mutations in the BRCA1 gene: c.5266dup (twenty-five samples), c.3607C>T (ten samples), and c.843_846del (five samples).

The variant c.5266dup (p.Gln1756ProfsTer74) found in the *BRCA1* gene results from the insertion/duplication of a single nucleotide G in the position 5266 in exon 19 and is associated with the SNP reference rs80357906. This variant, also known in the literature as c.5329dup (p.Gln1777ProfsTer74), 5382insC, or 5385insC, is considered to cause a frameshift mutation and has a molecular consequence (by changing the reading frame), leading to the appearance of a premature termination codon. This type of modification alters protein synthesis, leading to the formation of a truncated protein that has no C-terminal BRCT (BRCA1 C Terminus) domain or no protein result, causing a loss of function in the gene. The protein affected by this variant is unable to form nuclear foci, regardless of the higher concentration in the cytoplasm being directed to the nucleus by *BARD1* [34]. The heterozygous loss of function is a confirmed disease mechanism for the Hereditary Breast and Ovarian Cancer Syndrome. The ClinGen (Clinical Genome)-approved ENIGMA (Enhancing NeuroImaging Genetics through Meta-Analysis) panel classified this mutation as pathogenic in 2016 according to the American College of Medical Genetics (ACMG) criteria: PVS1, PP5, and PM2. This variant was shown to be a frequent founder mutation in the Ashkenazi Jewish population, and it has been reported in many individuals affected by breast or/and ovarian cancer all over the world, including Europe, Asia, America, and Africa, with a high prevalence in Poland and Eastern Europe, including Romania [35,36,37,38,39,40,41,42,43,44]. This variant has also been observed in patients with pancreatic or prostate cancer [45,46]. Carriers of this mutation were shown to have a higher risk of developing breast or ovarian cancer by the age of 70, and the risk for breast cancer is 67–89% and 22–42% for ovarian cancer [47,48,49]. The variant was reported in the ClinVar database with a pathogenic clinical significance (Variation ID: 17677) and is listed in various international databases: 0.000034 (TOPMED), 0.000183 (GnomAD_exome), 0.00040 (ALFA), and 0.000156 (ExAC). The Varsome clinical database classified this mutation as pathogenic, having a score of pathogenicity of 17 points.

The nonsense variant c.3607C>T (p.Arg1203Ter) found in the *BRCA1* gene results from the substitution of the nucleotide C to nucleotide T in the 3607 position in exon 10 and is associated with the SNP reference rs62625308. This variant, also known as c.3726C>T, is considered to cause a premature stop codon, therefore leading to a premature termination codon in position 1203 of the protein. The appearance of this stop codon determines the synthesis of a truncated protein or the absence of a protein. In the same way as the previous mutation, the heterozygous loss of function is a confirmed disease mechanism for the Hereditary Breast and Ovarian Cancer Syndrome. The ClinGen-approved ENIGMA panel classified this mutation as pathogenic in 2016, and the following ACMG criteria were applied: PVS1, PP5, and PM2. This variant has been reported in many individuals affected by breast and/or ovarian cancer of different cultural ethnicities [41,50,51,52,53,54,55,56]. It has been reported in ClinVar that this mutation has a pathogenic clinical significance (Variation ID: 17671) and it has the following frequencies in different databases: 0.000012 (GnomAD_exome), 0.000010 (ALFA), and 0.00001 (PAGE_STUDY). The Varsome clinical database classified this mutation as pathogenic, having a score of pathogenicity of 17 points.

The variant c.843_846del (p.Ser282TyrfsTer15) found in the *BRCA1* gene results from the deletion of four nucleotides (CTCA) at the position 843 to 846 in exon 10 and is associated with the SNP reference rs80357919. This variant, also known as 962del4, is considered to cause a frameshift mutation, leading to a premature termination codon, which determines a truncated protein or the lack of a protein. The loss of function in BRCA1 protein is a common mechanism for breast and ovarian cancer. The mutation has been observed in many individuals affected by breast and/or ovarian cancer [57,58,59,60,61,62,63]. The ENIGMA panel and 12 other submitters classified this mutation as pathogenic in 2014, and the following ACMG criteria were applied: PVS1, PP5, and PM2. It has been reported in ClinVar that this mutation has a pathogenic clinical significance (Variation ID: 17683), and its global frequency is not known. The Varsome clinical database classified this mutation as pathogenic, having a score of pathogenicity of 17 points.

In our study, we also identified four *BRCA1* VUS mutations: c.4843G>A (two samples), c.3711A>G (two samples), c.2666C>T (one sample), and c.994C>T (one sample). For the c.944C>T mutation, only computational prediction data were found with results that were inconclusive regarding the impact of this variant on protein structure and function. For the c.4843G>A mutation, along with computational predictions that were inconclusive, functional studies using *BRCA1*-deficient mouse embryonic stem cells in PARP inhibitor sensitivity assays have shown that this variant does not impact BRCA1 function in transcription activation [64]. For c.3711A>G and c.2666C>T mutations, the published data suggest that these alterations are not expected to disrupt BRCA1 protein function. All of these VUS mutations have been previously reported in individuals affected with breast and/or ovarian patients [65,66,67,68,69].

The most frequent pathogenic mutations in the BRCA2 gene that we identified in our data are c.4284dup (three samples), c.5379_5380ins (three samples), c.7180A>T (three samples), and c.9371A>T (three samples).

The variant c.4284dup (p.Gln1429SerfsTer9) found in the *BRCA2* gene results from the insertion/duplication of a single nucleotide T in the position 4284 in exon 11 and is associated with SNP reference rs80359439. This mutation, also known as 4512dupT or 4510insT, has a frameshift molecular consequence (changing the reading frame), which means that instead of the Glutamine amino acid, it determines the formation of a premature termination codon that leads to the synthesis of a truncated protein or the degradation of the protein, thus altering the protein function. The variant was classified by the ClinGen-approved ENIGMA as a pathogenic mutation, in accordance with the following ACMG criteria: PVS1, PP5, and PM2. It has been observed in many patients with breast and/or ovarian cancer [60,70,71,72,73,74]. This mutation was also described in one patient with medulloblastoma and in another patient with prostate cancer and other malignancies [75,76]. It has been reported in ClinVar that this mutation has a pathogenic clinical significance (Variation ID: 37892), and it has the following frequencies in different databases: 0.000015 (TOPMED) and 0.000004 (GnomAD_exome). The Varsome clinical database classified this mutation as pathogenic, having a score of pathogenicity of 17 points.

The variant c.5379_5380insTT (p.Val1794LeufsTer2) results from the insertion of two nucleotides T in positions 5379_5380 in exon 11 and is associated with SNP reference rs879255329. This is a frameshift type of mutation that alters the open reading frame, creating a premature stop-codon that disrupts the translation of two amino acids thereafter. This interruption results in a truncated protein or no protein at all. This variant is classified as pathogenic by ClinVar (Variation ID: 37892) and Varsome Clinical (having a score of pathogenicity of 17 points), and its global frequency is unknown.

The variant c.7180A>T (p.Arg2394Ter) results from the substitution of an A to T in the 7180 position in exon 13 and is associated with SNP reference rs80358946. This mutation, also known as 7408A>T, has a gained stop codon, which leads to the loss of function of the protein because of its truncated conformation. It has been reported in many affected individuals with breast and/or ovarian cancer [77,78,79,80,81]. This variant is classified as pathogenic in ClinVar (Variation ID: 52279) and Varsome Clinical (having a score of pathogenicity of 17 points), and its global frequency in GnomAD is 0.000007.

The variant c.9371A>T (p.Asn3124Ile) results from the substitution of A to T in the 9371 position in exon 24 and is associated with SNP reference rs28897759. This variant is a missense mutation that leads to the replacement of the asparagine amino acid with isoleucine in the 3124 position. This modification affects not only the conformation of the DNA binding domain but also the functionality of the protein [82,83,84,85,86]. The variant was described in individuals affected by breast and/or ovarian cancer [87,88,89,90] and was shown to be a frequently found mutation in patients from Germany, Poland, and Romania [35,41,87,91]. This variant is classified as pathogenic in ClinVar (Variation ID: 38233) and Varsome Clinical (having a score of pathogenicity of 16 points) and has the following global frequencies: 0.000007 (gnomAD) and 0.000004 (TopMed).

We also identified eight BRCA2 VUS mutations: c.4412_4414del (two samples), and c.3070A>G, c.191C>T, c.7007+70T>G, c.442T>C, c.2396A>G, c.4446_4451dup, and c.8254A>T were found in only one sample.

For c.3070A>G, c.191C>T, c.442T>C, c.2396A>G, and c.8254A>T mutations, the studies show that the sequence alterations are not expected to disrupt BRCA2 protein function. The c.4412_4414del and c.4446_4451dup mutations do not have, to our knowledge, any data from experimental studies or algorithm predictions [92]. All these variants have been previously reported in individuals with breast and/or ovarian cancer [58,93,94,95,96].

## 5. Conclusions

Our investigation revealed the most common mutations in the *BRCA1* and *BRCA2* genes associated with breast and ovarian cancer. Our data also support that the c.5266dup mutation in the *BRCA1* gene could be considered a founder mutation in Eastern Europe, as previously described in the literature. Because of the recent studies that show the implications of mutations from other predisposing genes (e.g., *PALB2, CHEK2, ATM, RAD51C, RAD51D, BRIP1,* etc.) in these types of cancers and also the association of the *BRCA1* and *BRCA2* mutations in other types of cancers, it is necessary to extend the panel of genes tested and the types of cancer (e.g., pancreatic cancer, prostate cancer). We consider that the results of our study provide data regarding *BRCA1* and *BRCA2* mutations to create the genetic database needed for the Romanian population.

## Figures and Tables

**Table 1:** Clinical and demographic characteristics of the patients included in our group study.

		Patients with Non-Mucinous Ovarian Cancer, High-Grade Serous Ovarian Cancer Subtype (n = 390)	Patients with Non-Mucinous Ovarian Cancer with No Defined Subtype (n = 42)	Patients with Triple Negative Breast Cancer (n = 184)
Sex-Female		all female patients	all female patients	all female patients
Age in years, average (range)		60 years old (25–87)	61 years old (34–81)	58 years old (28–81)
Number of patients from each region of the country	Transilvania	93	12	38
	Moldova	109	12	48
	Dobrogea	16	2	12
	Muntenia	144	9	73
	Oltenia	28	7	13
Number of patients with negative results		300	30	152
Number of patients with pathogenic *BRCA1* mutations		48	3	18
Number of patients with pathogenic *BRCA2* mutations		24	6	4
Number of patients with VUS *BRCA1* mutations		2	0	2
Number of patients with VUS *BRCA2* mutations		6	1	2
Number of patients with c.9976A>T *BRCA2* mutation		5	2	4
Number of patients with large deletions in the *BRCA1* gene		5	0	2

**Table 2:** The pathogenic mutations in the *BRCA1* gene found in our group study.

HGVS Nomenclature *	The rs Code	Ref	Alt	Gene Position	Protein Change	Consequence	Allele Frequency	Number of Samples That Have This Mutation
c.3700_3704del	rs80357609	GTAAA	-	exon 10	p.Val1234fs	frameshift	0.00000 (0/10680, ALFA); 0.000004 (1/264690, TOPMED)	2 (1A + 1C)
c.213-12A>G	rs80358163	T	C	intron 4	-	intron variant	0.00006 (1/16810, ALFA)	1 (1A)
c.3607C>T	rs62625308	G	A	exon 10	p.Arg1203Ter	stop gained/nonsense	0.000010 (1/100184, ALFA); 0.000012 (3/251014, GnomAD_exome)	10 (8A + 1B + 1C)
c.2955del	rs397509027	C	-	exon 10	p.Ile986fs	frameshift	-	1 (1A)
c.5266dup	rs80357906	-	G	exon 20	p.Gln1756fs	frameshift	0.00040 (11/27508; ALFA); 0.000183 (46/251494, GnomAD_exome)	25 (16A + 2B + 7C)
c.342del	rs886040129	A	-	exon 6	p.Pro115fs	frameshift	-	1 (1A)
c.181T>G	rs28897672	A	C	exon 4	p.Cys61Gly	missense	0.00000 (0/16646, ALFA); 0.000032 (8/250754, GnomAD_exome)	4 (2A + 2C)
c.1687C>T	rs80356898	G	A	exon 10	p.Gln563Ter	stop gained/nonsense	0.000000 (0/163772, ALFA); 0.000007 (1/140138, GnomAD)	3 (2A + 1C)
c.1961del	rs80357522	T	-	exon 10	p.Lys654fs	frameshift	0.00007 (1/14050, ALFA); 0.000008 (2/264690, TOPMED)	1 (1A)
c.3756_3760del	rs886040164	TAGAC	-	exon 10	p.Ser1253fs	frameshift	-	1 (1A)
c.5328_5329insC	rs80357751	-	G	exon 21	p.Thr1777fs	frameshift	-	3 (3A)
c.4035del	rs80357711	T	-	exon 10	p.Glu1346fs	frameshift	0.00037 (6/16332, ALFA); 0.000020 (5/251112, GnomAD_exome)	4 (4A)
c.1450G>T	rs80357304	C	A	exon 10	p.Gly484Ter	stop gained/nonsense	0.00000 (0/11158, ALFA)	1 (1A)
c.4689C>G	rs80357433	G	C	exon 16	p.Tyr1563Ter	stop gained/nonsense	0.00000 (0/14050, ALFA); 0.000004 (1/250704, GnomAD_exome)	2 (1A + 1C)
c.843_846del	rs80357919	TGAG	-	exon 10	p.Ser282fs	frameshift	-	5 (3A + 2C)
c.3228_3229del	rs80357635	AG	-	exon 10	p.Gly1077fs	frameshift	0.000004 (1/250376, GnomAD_exome)	2 (1A + 1C)
c.2155A>T	rs80357147	T	A	exon 10	p.Lys719Ter	stop gained/nonsense	0.00027 (4/14710, ALFA); 0.000040 (10/250994, GnomAD_exome)	1 (1A)
c.5314C>T	rs80357123	G	A	exon 20	p.Arg1751Ter	stop gained/nonsense	0.00000 (0/14526, ALFA); 0.000012 (3/251492, GnomAD_exome)	1 (1C)
c.2511del	rs1555589501	A	-	exon 10	p.Asn838fs	frameshift	-	1 (1C)

* HGVS nomenclature (DNA variants are enumerated according to NCBI reference sequence; the *-* symbol in the frequency columns represents the fact that in those databases, we did not find frequencies for these variants “https://www.ncbi.nlm.nih.gov (accessed on 3 October 2023)”; A—patients with non-mucinous ovarian cancer, high-grade serous ovarian cancer subtype; B—patients with non-mucinous ovarian cancer with no defined subtype; C—triple-negative breast cancer.

**Table 3:** The mutations in the *BRCA1* gene with unknown significance (VUS) found in our group study.

HGVS Nomenclature *	The rs Code	Ref	Alt	Gene Position	Protein Change	Consequence	Allele Frequency	Number of Samples That Have This Mutation
c.2666C>T	rs769712441	G	A	exon 10	p.Ser889Phe	missense variant	0.00000 (0/10680, ALFA); 0.000012 (3/251042, GnomAD_exome)	1 (1A)
c.4843G>A	rs80356987	C	T	exon 16	p.Ala1615Thr	missense variant	0.00004 (1/23038, ALFA); 0.000004 (1/251402, GnomAD_exome)	2 (1A + 1C)
c.3711A>G	rs80357388	T	C	exon 10	p.Ile1237Met	missense variant	0.00004 (1/23038, ALFA); 0.000004 (1/251284, GnomAD_exome)	2 (1A + 1C)
c.994C>T	rs80357176	G	A	exon 10	p.Arg332Trp	missense variant	0.00006 (2/35426, ALFA); 0.000008 (2/251342, GnomAD_exome)	1 (1C)

* HGVS nomenclature (DNA variants are enumerated according to NCBI reference sequence; the *-* symbol in the frequency columns represents the fact that in those databases, we did not find frequencies for these variants “https://www.ncbi.nlm.nih.gov (accessed on 3 October 2023)”; A—patients with non-mucinous ovarian cancer, high-grade serous ovarian cancer subtype; B—patients with non-mucinous ovarian cancer with no defined subtype; C—triple-negative breast cancer.

**Table 4:** The pathogenic mutations in the ***BRCA2*** gene found in our group study.

HGVS Nomenclature *	The rs Code	Ref	Alt	Gene Position	Protein Change	Consequence	Allele Frequency	Number of Samples That Have This Mutation
c.4284dup	rs80359439	-	T	exon 11	p.Gln1429fs	frameshift	0.00000 (0/14050, ALFA); 0.000004 (1/244426, GnomAD_exome)	3 (2A + 1B)
c.9371A>T	rs28897759	A	T	exon 25	p.Asn3124Ile	missense	0.00000 (0/14050, ALFA); 0.000007 (1/139834, GnomAD)	3 (1A + 2C)
c.3545_3546del	rs80359388	TT	-	exon 11	p.Gln1181_Phe1182insTer	stop gained	0.00000 (0/14050, ALFA); 0.000020 (5/250970, GnomAD_exome)	1 (1A)
c.7180A>T	rs80358946	A	T	exon 14	p.Arg2394Ter	stop gained	0.000007 (1/140286, GnomAD)	3 (2A + 1B)
c.1796_1800del	rs276174813	CTTAT	-	exon 10	p.Ser599Terfs	stop gained	0.000004 (1/244054, GnomAD_exome)	1 (1A)
c.475+1G>T	rs81002797	G	T	intron 5	-	splice donor	-	1 (1A)
c.4666del	-	A	-	exon 11	p.Ile1556SerfsTer12	frameshift	-	1 (1A)
c.5379_5380ins	rs879255329	-	TT	exon 11	p.Val1794fs	frameshift	-	3 (3A)
c.5946del	rs80359550	T	-	exon 11	p.Ser1982fs	frameshift	0.00074 (17/23038, ALFA); 0.000291 (73/250700, GnomAD_exome)	2 (2A)
c.3195_3198del	rs80359375	TAAT	-	exon 11	p.Asn1066fs	frameshift	-	1 (1A)
c.7878G>C	rs80359013	G	C	exon 17	p.Trp2626Cys	stop gained	0.00003 (1/36092, ALFA); 0.000008 (2/251276, GnomAD_exome)	1 (1A)
c.2197_2198ins	-	-	G	exon 11	p.Val733Glyfs*22	frameshift	-	1 (1A)
c.6267_6269delinsC	rs276174868	delGCAinsC		exon 11	p.Glu2089fs	frameshift	-	1 (1A)
c.5279C>G	rs80358751	C	G	exon 11	p.Ser1760Ter	stop gained	0.00000 (0/78700, PAGE_STUDY)	1 (1A)
c.5073dup	rs80359479	-	A	exon 11	p.Trp1692fs	frameshift	0.00014 (2/14050, ALFA); 0.000017 (4/230778, GnomAD_exome)	1 (1A)
c.4964dup	rs398122789	-	A	exon 11	p.Tyr1655Terfs	stop gained	0.00000 (0/10680, ALFA);	2 (1A + 1B)
c.7617+2T>G	rs81002843	T	G	intron 15	-	splice donor	0.000 (0/660, ALFA); 0.00001 (1/78700, PAGE_STUDY)	1 (1A)
c.4987_4990del	rs397507753	GTCA	-	exon 11	p.Val1663fs	frameshift	-	1 (1A)
c.3847_3848del	rs80359405	GT	-	exon 11	p.Val1283fs	frameshift	0.00000 (0/14050, ALFA); 0.000054 (11/204502, GnomAD_exome)	1 (1A)
c.3975_3978dup	rs397515636	-	TGCT	exon 11	p.Ala1327TrpfsTer4	frameshift	0.00000 (0/10680, ALFA); 0.000010 (1/103324, ExAC)	2 (2B)
c.5576_5579del	rs80359520	TTAA	-	exon 11	p.Ile1859fs	frameshift	0.00000 (0/14050, ALFA); 0.000016 (4/244832, GnomAD_exome)	1 (1B)
c.9097dup	rs397507419	-	A	exon 23	p.Thr3033fs	frameshift	0.00000 (0/14050, ALFA); 0.000007 (1/139854, GnomAD)	1 (1C)
c.5162del	rs1555284090	A	-	exon 11	p.Asn1721fs	frameshift	-	1 (1C)

* HGVS nomenclature (DNA variants are enumerated according to NCBI reference sequence; the *-* symbol in the frequency columns represents the fact that in those databases, we did not find frequencies for these variants “https://www.ncbi.nlm.nih.gov (accessed on 3 October 2023)”; A—patients with non-mucinous ovarian cancer, high-grade serous ovarian cancer subtype; B—patients with non-mucinous ovarian cancer with no defined subtype; C—triple-negative breast cancer.

**Table 5:** The mutations in the ***BRCA2*** gene with unknown significance (VUS) found in our group study.

HGVS Nomenclature *	The rs Code	Ref	Alt	Gene Position	Protein Change	Consequence	Allele Frequency	Number of Samples That Have This Mutation
c.4412_4414del	rs886039317	GAA	-	exon 11	p.Arg1471del	inframe deletion	-	2 (1A + 1C)
c.3070A>G	rs876659687	A	G	exon 11	p.Ile1024Val	missense	0.00000 (0/10680, ALFA)	1 (1A)
c.191C>T	rs397507615	C	T	exon 3	p.Thr64Ile	missense	0.000004 (1/251382, GnomAD_exome)	1 (1A)
c.7007+70T>G	-	T	G	intron 16	-	splice donor	-	1 (1A)
c.442T>C	rs80358677	T	C	exon 5	p.Cys148Arg	missense	0.00005 (2/41050, ALFA); 0.000007 (1/140270, GnomAD)	1 (1A)
c.2396A>G	rs1555282656	A	G	exon 11	p.Lys799Arg	missense	0.00006 (1/16760, 3.5KJPNv2)	1 (1A)
c.4446_4451dup	rs863224826	-	AACAGA	exon 11	p.Glu1482_Thr1483dup	inframe insertion	0.000004 (1/249934, GnomAD_exome)	1 (1B)
c.8254A>T	rs80359072	A	T	exon 18	p.Ile2752Phe	missense	-	1 (1C)

* HGVS nomenclature (DNA variants are enumerated according to NCBI reference sequence; the *-* symbol in the frequency columns represents the fact that in those databases, we did not find frequencies for these variants “https://www.ncbi.nlm.nih.gov (accessed on 3 October 2023)”; A—patients with non-mucinous ovarian cancer, high-grade serous ovarian cancer subtype; B—patients with non-mucinous ovarian cancer with no defined subtype; C—triple-negative breast cancer.

## Data Availability

If needed, we will find a way to provide further information.
